# X-ray Digital Radiography and Computed Tomography

**DOI:** 10.3390/jimaging8050119

**Published:** 2022-04-21

**Authors:** Maria Pia Morigi, Fauzia Albertin

**Affiliations:** 1 Department of Physics and Astronomy “Augusto Righi”, University of Bologna, Viale Carlo Berti Pichat 6/2, 40127 Bologna, Italy; 2 Istituto di Scienze e Tecnologie Chimiche “Giulio Natta” SCITEC-CNR, Via dell’ Elce di Sotto 8, 06123 Perugia, Italy; fauzia.albertin@gmail.com

In recent years, X-ray imaging has rapidly grown and spread beyond the medical field; today, it plays a key role in diverse research areas. The potential and non-invasiveness of X-ray digital radiography and computed tomography have made X-ray imaging a fundamental tool in different fields, from medical diagnostics to the characterization of materials and industrial components, as well as cultural heritage investigations.

The design and development of new detectors and new X-ray sources, combined with the increased computing power, have made it possible to achieve previously unthinkable results in terms of image quality and spatial resolution, enabling the imaging of even submicron details with lab-based instrumentation.

The aim of this Special Issue, “X-ray Digital Radiography and Computed Tomography”, is to present and highlight novel instrumentation and cutting-edge methods for X-ray imaging, as well as different applications in various fields.

This Special Issue received submissions focused on the most diverse research areas and coming from several different countries—from Europe to the US and Australia. After a rigorous review process, 23 manuscripts were selected—19 research articles and 4 review papers—illustrating the applications of X-ray digital radiography and computed tomography in many sectors, from medical imaging [1,2,3] to the investigation of cultural heritage objects [4,5,6], combining X-ray and neutron imaging [7].

Further topics covered in this Special Issue include the use of synchrotron light for phase-contrast and multi-contrast imaging [8,9,10,11] and analytical techniques, such as microscopic synchrotron X-ray fluorescence [12]. Several contributions deal with new performing algorithms developed for the suppression of cone-beam artifacts in CT images [13] and signal retrieval from non-sinusoidal intensity modulations in X-ray and neutron interferometry [14], or devoted to innovative techniques, such as multiscale holotomography [15], rotation-free dynamic multi-angle X-ray tomography [16] and high-speed X-ray imaging [17]. The last two research papers describe the implementation principles of a complete CT reconstruction toolchain [18] and the design and realization of a new furnace for in situ wettability experiments at high temperatures under X-ray microtomography [19].

Interestingly, four review papers illustrate the principles and perspective of radiographic imaging with muons [20], an overview of photon-counting spectral imaging detectors [21], a comparison of imaging techniques for biological and biomedical studies [22] and a review of the use of coherent X-rays, from early synchrotron tests to the most recent brain studies [23].

As the Special Issue Editors, we hope that this book will benefit the scientific community and contribute to the spread of knowledge of the numerous and diversified applications of X-ray digital radiography and computed tomography.

We would like to take this opportunity to thank all the authors for their contributions, the reviewers for the efforts to enhance the quality of the manuscripts, and the MDPI editorial team for their assistance in making our editorial tasks easier.
